# EVALUATION OF THE PERFORMANCE OF CHATGPT/ARTIFICIAL INTELLIGENCE IN THE MULTIPLE-CHOICE TEST TO OBTAIN THE TITLE OF SPECIALIST IN ORTHOPEDICS AND TRAUMATOLOGY

**DOI:** 10.1590/1413-785220243201e280947

**Published:** 2025-04-07

**Authors:** LUCAS PLENS DE BRITTO COSTA, DANILO HENRIQUE PIZZO DE CASTRO, RENATO PINHEIRO CORDEIRO, RÔMULO BALLARIN ALBINO

**Affiliations:** 1 Universidade Estadual Paulista, Grupo de Medicina e Cirurgia do Pe e Tornozelo, Department of Surgery and Orthopedics, Botucatu, SP, Brazil.; 2 Universidade Federal de São Paulo, Escola Paulista de Medicina, Departamento de Ortopedia e Traumatologia, São Paulo,SP, Brazil.

**Keywords:** Artificial Intelligence, Orthopedics, Medical Education, Inteligência Artificial, Ortopedia, Educação Médica

## Abstract

**Introduction:**

ChatGPT, an advanced Artificial Intelligence model specialized in natural language processing, shows remarkable abilities, achieving high scores in certification exams in various specialties. This study aims to evaluate ChatGPT’s performance in multiple-choice tests applied to obtain specialist certification in Orthopedics and Traumatology.

**Methods:**

We used ChatGPT 4.0 to answer 100 questions from the first phase of the *Título de Especialista em Ortopedia e Traumatologia* 2022 (TEOT) (Specialist in Orthopedics and Traumatology Test). We excluded non-text-based questions. Each question was entered individually into ChatGPT, with a new session initiated for each question. Performance was evaluated regarding number of words and questions’ taxonomic classification.

**Results:**

Of the 95 questions analyzed, ChatGPT answered 61.05% correctly and 38.95% incorrectly. There was no statistically significant difference regarding number of words, and ChatGPT’s performance did not vary according to taxonomic level.

**Conclusion:**

ChatGPT demonstrated vast knowledge in Orthopedics, with acceptable performance in the TEOT exam. Results suggest ChatGPT’s an educational and clinical resource in Orthopedics, but needs future progress and human supervision for its effective application. ***Level of evidence IV, Case series.***

## RESUMO

O ChatGPT, um avançado modelo de Inteligência Artificial especializado em processamento de linguagem natural, tem mostrado habilidades notáveis, alcançando pontuações altas em exames de certificação em várias especialidades. Este estudo foi conduzido com o objetivo de avaliar o desempenho do ChatGPT no teste de múltipla escolha aplicado para se obter a certificação de especialista em Ortopedia e Traumatologia.

**Métodos:**

Utilizamos o ChatGPT 4.0 para responder 100 perguntas da primeira fase do TEOT 2022. Excluímos perguntas não baseadas em texto. Cada questão foi inserida individualmente no ChatGPT, com uma nova sessão iniciada para cada questão. A performance foi avaliada em relação ao número de palavras e à classificação taxonômica das questões.

**Resultados:**

Das 95 questões analisadas, o ChatGPT respondeu corretamente 61.05% e incorretamente 38.95%. Não houve diferença estatística significativa em relação ao número de palavras das questões e o ChatGPT não apresentou variação de desempenho conforme o nível taxonômico.

**Conclusão:**

O ChatGPT demonstrou vasto conhecimento em ortopedia, com um desempenho aceitável no exame TEOT, resultado que sugere o potencial do ChatGPT como recurso educacional e clínico em ortopedia, porém com a necessidade de progressos futuros e supervisão humana para sua aplicação efetiva. **Nível de evidência IV, Série de casos.**

## INTRODUCTION

Over the last ten years, Artificial Intelligence (AI) revolutionized the way we perform tasks in different fields, ranging from medical sciences to finance and administration.^1^ ^)-(^ ^4^ In this context, ChatGPT, developed by OpenAI, stands out in the scope of advanced natural language processing. It is part of the Large Language Model (LLM) and based on data, generating natural language responses and adapting them to conversational contexts. Confined to a server environment, ChatGPT works with pre-existing information, without the ability to search for new data or perform up to date research. Its ability to develop answers comes from an abstract analysis of connections between words in its neural network, a different technique than those employed by conventional chatbots, which access online databases and have additional informational resources. ^(^ ^5^ ^),(^ ^6^ AI emerged as a tool in medical education and quick access to data, ranging from computer models to virtual reality simulators and adaptive learning platforms. ^(^ ^7^ ^)-(^ ^9^ In Brazil, Orthopedics is one of the fields that most require certification from the Brazilian Society of Orthopedics and Traumatology. The certification process includes theoretical and practical exams, analysis of clinical cases and other criteria. The multiple-choice test is challenging and requires extensive knowledge in Orthopedics. ^(^ ^10^

This study’s main goal is to evaluate what percentage of questions in the first phase of the *Título de Especialista em Ortopedia e Traumatologia* (TEOT) (Specialist in Orthopedics and Traumatology) exam can be answered correctly by ChatGPT. Secondary goals include investigating the influence of number of words on ChatGPT’s accuracy and a correlation between questions’ taxonomic classification and accuracy of responses provided by ChatGPT.

## METHODS

This was an experimental study using a commercial LLM (ChatGPT 4.0). ^5^ The multiple-choice test from the first phase of TEOT 2022, with 100 publicly available questions, was selected. Questions containing non-text-based data were excluded. The provided answers were compared with the official template, also publicly available. ^(^ ^10^

**Figure 1:**
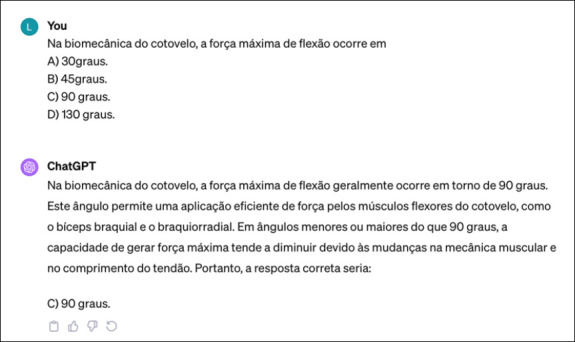
Example of ChatGPT’s answers.

All questions were individually entered into ChatGPT’s text box as originally written, including answer options. To reduce memory retention bias, a new session was initiated for each question (Figure 1).

If ChatGPT did not select an answer or expressed more than one correct answer, the question was re-entered with the command “select the best answer.” If ChatGPT did not select an answer by the second request, the question was listed as “did not answer” and the next question was provided.

### ChatGPT’s performance according to number of words in the questions

For each question, the number of words was provided by the Pages app (Apple) with the word counting tool, excluding the question number and punctuation marks.

### ChatGPT’s performance according to question taxonomy

To verify ChatGPT’s performance concerning increasingly challenging levels of question taxonomy, two board-certified orthopedists classified the questions according to Buckwalter’s taxonomic scheme. ^(^ ^11^ Questions were divided into three groups: type 1 tests recognition and recall only, type 2 assesses comprehension and interpretation, and type 3 asks about application of knowledge.

### Statistical Analysis

Questions’ data were compared through quantitative statistical analysis to determine main differences in terms of number of words. The Shapiro-Wilk test was used for all data, and the Wilcoxon test was used when normality was rejected. Pearson’s chi-squared test verified whether the percentage of ChatGPT’s correct and incorrect answers varied according to questions’ taxonomic classification. Statistical tests were performed using the R software (version 4.0.3) with a significance level of 5%.

## RESULTS

### Percentage of questions answered correctly

A total of 95 questions were analyzed, excluding five questions due to images, and ChatGPT was able to answer all questions, regardless of assertiveness. ChatGPT answered 61.05% correctly (58/95 questions) and 38.95% incorrectly (37/95 questions).

### ChatGPT’s performance regarding number of words

Among questions answered incorrectly, the mean word count was 18.42, ranging from 8 to 28, with a standard deviation of 5.22. Among questions answered correctly, the average word count was 17.93, ranging from 8 to 37, with a standard deviation of 5.47. There was no statistical difference in number of words between questions answered correctly or incorrectly (p = 0.660, 95%CI). (Table 1).

**Table 1:** Word Count x Assertiveness

Word Count	Average	Min - Max	Standard deviation	P
Correct Answers	17.94	8 - 37	5.47	0,6411
Incorrect Answers	18.45	8 - 28	5.29	

### ChatGPT’s performance regarding questions’ taxonomic complexity

Of the 95 questions evaluated, 56 were classified as type 1, 39 were classified as type 2 and none were classified as type 3. ChatGPT’s performance did not change regarding questions’ taxonomic level, correctly answering 34 of the 56 type-1 questions (60.71%) and 24 of the 39 type-2 questions (61.53%), with no statistically significant difference (p = 0.9354, 95%CI) (Table 2).

**Table 2:** Taxonomic Classification vs Assertiveness

Taxonomic Classification	Correct Answers	Incorrect Answers	P
Type 1	34	22	0.9354
Type 2	24	15	

## DISCUSSION

ChatGPT, a state-of-the-art language model developed by OpenAI, showed remarkable achievements in various domains. ^1^ ^),(^ ^12^ Although a higher standard should be set for it to gain credibility as an educational or clinical decision-making tool, its current performance and rapid improvement suggest this standard may be viable in due course. ^(^ ^13^ We sought to determine whether ChatGPT could be used similarly for orthopedic residents by determining its competence in the field using TEOT.

In our study, we evaluated ChatGPT’s performance in the first phase of TEOT 2022 and it performed well enough to pass the multiple-choice phase, with assertiveness above the 60% mark, regardless of number of words or taxonomic classification of questions. 

Many studies analyzed ChatGPT’s performance in training and certification tests in medical specialties. ChatGPT achieved a performance equivalent to a first-year resident on the UK Plastic Surgery Examination, answering about 55% of questions correctly. ^14^ ^)-(^ ^15^

In Orthopedics, Lum and colleagues examined ChatGPT’s performance on Orthobullets (Lineage Medical) practical questions, noting the system answered 47% of questions correctly. They noticed a variation in accuracy, which decreased as taxonomic complexity of questions increased. ^6^ Kung and collaborators ^16^ evaluated the performance of ChatGPT 4.0 on the Orthopedic In-Training Examination between 2020 and 2022, finding an average of 73.6% correct answers, which matches the average performance of a fifth-year resident and exceeds the corresponding passing score for the American Board of Orthopaedic Surgery Part I.

We have not identified studies correlating number of words with ChatGPT’s assertiveness. However, OpenAI claims their artificial intelligence model can process up to 25,000 words with accuracy and contextualization. ^(^ ^5^

After analyzing ChatGPT’s incorrect answers, we identified possible conflicting information sources on different topics. This can hinder ChatGPT’s ability to answer questions correctly. In addition, ChatGPT 4.0 is only trained with information up to April 2023, so new information used in medical tests may not be available. Specifically in Medicine, there can be multiple potentially correct answers to a question with only one best answer, which can be challenging for AI if there is correct information supporting each answer. A potential solution would be to train an AI model with only peer-reviewed medical literature, such as PubMed. ^(^ ^14^

Despite these results, there are several limitations to our study. First, the current version of ChatGPT cannot analyze images, making it difficult to evaluate an essential skill for orthopedic surgeons. However, given the rapid progress in AI learning, we anticipate future models will incorporate image analysis. We also observed cases where ChatGPT provided a verifiable source of information, but still gave an incorrect answer, citing articles that were outdated or showed little evidence, or drew incorrect conclusions based on certain sentences that did not represent their conclusions. These logical errors based on false or incomplete facts are worrying and were even defined as the “hallucination effect,” to which ChatGPT is susceptible. ^16^ Finally, our study did not present questions with level-3 taxonomy, which require a higher degree of interpretation and application of data that could affect ChatGPT’s assertiveness.

## CONCLUSION

Due to the evolution of standards established by AI, it is important that orthopedic professionals actively incorporate this technology, steering it towards its application in providing patient care. ChatGPT, in its current configuration, shows vast knowledge in Orthopedics, and with progress, under human supervision, it will play a relevant role in medical training, patient instruction and clinical decisions.
